# Persistence of *Salmonella enterica* and *Enterococcus faecium* NRRL B-2354 on Baby Spinach Subjected to Temperature Abuse after Exposure to Sub-Lethal Stresses

**DOI:** 10.3390/foods10092141

**Published:** 2021-09-10

**Authors:** Zhao Chen, Jianghong Meng

**Affiliations:** 1 Joint Institute for Food Safety and Applied Nutrition, University of Maryland, College Park, MD 20742, USA; zhchen29@umd.edu; 2 Center for Food Safety and Security Systems, University of Maryland, College Park, MD 20742, USA; 3 Department of Nutrition and Food Science, University of Maryland, College Park, MD 20742, USA

**Keywords:** *Salmonella enterica*, leafy green, temperature abuse, sub-lethal stress, cross-protection, surrogate, mathematical modeling

## Abstract

The exposure of foodborne pathogens such as *Salmonella enterica* to a sub-lethal stress may protect bacterial cells against distinct stresses during the production of leafy greens, which can constitute potential health hazards to consumers. In this study, we evaluated how the prior exposure of *S. enterica* to sub-lethal food processing-related stresses influenced its subsequent persistence on baby spinach under cold (4 °C for 7 days) and temperature abuse (37 °C for 2 h + 4 °C for 7 days) conditions. We also compared the survival characteristics of pre-stressed *S. enterica* and *Enterococcus faecium* NRRL B-2354 as its surrogate on baby spinach. A cocktail of three *S. enterica* serovars, as well as *S.* Typhimurium ATCC 14028 wild type and its Δ*rpoS* mutant, and *E. faecium* NRRL B-2354, was first exposed to sub-lethal desiccation, oxidation, heat shock, and acid stresses. Afterward, baby spinach was inoculated with unstressed or pre-stressed cells at 7.0 log CFU/sample unit, followed by 7-day storage under cold and temperature abuse conditions. The unstressed *S. enterica* (fresh cells in sterile 0.85% saline) decreased rapidly within the first day and thereafter persisted around 5.5 log CFU/sample unit under both conditions. The desiccation-stressed *S. enterica* showed the highest bacterial counts (*p* < 0.05) compared to other conditions. The unstressed *S. enterica* survived better (*p* < 0.05) than the oxidation- and acid-stressed *S. enterica*, while there were no significant differences (*p* > 0.05) between the unstressed and heat-shocked *S. enterica*. Unlike the wild type, temperature abuse did not lead to the enhanced survival of the Δ*rpoS* mutant after exposure to desiccation stress, indicating that the *rpoS* gene could play a critical role in the persistence of desiccation-stressed *S. enterica* subjected to temperature abuse. *E. faecium* NRRL B-2354 was more persistent (*p* < 0.05) than the pre-stressed *S. enterica* under both conditions, suggesting its use as a suitable surrogate for pre-stressed *S. enterica* by providing a sufficient safety margin. Our results demonstrate the merit of considering the prior exposure of foodborne pathogens to sub-lethal stresses when validating the storage conditions for leafy greens.

## 1. Introduction

As estimated by the Centers for Disease Control and Prevention (CDC), *Salmonella enterica* causes about 1.35 million infections, 26,500 hospitalizations, and 420 deaths in the United States each year [1]. *S. enterica* has been implicated in many foodborne outbreaks and recalls linked to the consumption of leafy greens such as baby spinach [2,3]. Foodborne pathogens such as *S. enterica* may undergo physiological adaptations to a wide range of harsh environmental stresses encountered in various ecological niches and the earlier stages of food production [4], which could induce cross-protection towards subsequent processes [5,6]. Prior exposure to sub-lethal stresses may, thus, nullify the benefits of control efforts beyond these stages. Moreover, pre-stressed pathogens that contaminate leafy greens at both preharvest and postharvest levels can be better suited to persist and even proliferate or become more virulent [7]. The stress responses in bacteria are controlled by master regulators, which include alternative sigma factors such as RpoS (σ^S^) encoded by the *rpoS* gene [8]. Previous research has demonstrated that the *rpoS* gene is involved in the cross-protection of pre-stressed *S. enterica* against subsequent lethal stresses [6,9].

The leaf surface is considered a hostile environment, where bacteria encounter multiple stresses [10]. Unstressed cells from fresh cultures may not accurately reflect their survival characteristics on leafy greens under realistic conditions. Although the survival of foodborne pathogens such as *S. enterica* on leafy greens subjected to temperature abuse has been extensively studied [11,12,13], much less is known about their ability to develop cross-protective responses to the circumstances on leaf surfaces after exposure to sub-lethal food processing-related stresses. Hence, there is merit in evaluating food systems by considering them as stressful environments. Samelis and Sofos [14] stated that challenge studies should be based on worst-case scenarios by using cells that have been exposed to conditions as stressful as they are expected to encounter on foods. As also recommended by the United States Department of Agriculture (USDA) [15], the adaptation of cells for challenge studies should mimic the possible physiological state of the microorganism when it contaminates the food. It is, therefore, necessary to reconsider that challenge studies should be designed by introducing cells pre-exposed to sub-lethal stresses commonly encountered by pathogens in the food production systems.

Consistently maintaining adequate refrigeration during the storage of leafy greens is important for microbial food safety. The Sanitary Transportation of Human and Animal Food under the Food Safety Modernization Act (FSMA) emphasizes temperature control as a critical factor to minimize microbial food safety hazards [16]. Likewise, the current U.S. Food Code also requires that Time/Temperature Control for Safety (TCS) foods such as leafy greens should be maintained at 5 °C or below [17]. Nevertheless, temperature abuse may still occur during the cold storage of leafy greens. The increasing evidence of pathogen persistence on leafy greens under the temperature abuse condition brings in the question of how pre-stressed pathogens persist on leafy greens subjected to abusive temperatures.

Surrogates are non-pathogenic proxies for the pathogen of concern that have a comparable or more robust survival capability, which can be used to predict those of the target pathogen under the conditions being studied [18]. *Enterococcus faecium* NRRL B-2354 has been identified as a suitable *S. enterica* surrogate for validating the thermal processes of almonds by the Almond Board of California [19]. Additionally, *E. faecium* NRRL B-2354 may be used as a surrogate for alternative processes, but additional data should be gathered to support such applications. *E. faecium* NRRL B-2354 has not been used as a surrogate for *S. enterica* during the storage of leafy greens. Moreover, it is still unproven whether *E. faecium* NRRL B-2354 can serve as a surrogate for *S. enterica* pre-exposed to sub-lethal stresses when evaluating food storage scenarios.

In this work, we evaluated the impact of the prior exposure of *S. enterica* to a sub-lethal food processing-related stress (desiccation, oxidation, heat shock, or acid stress) on its subsequent persistence on baby spinach under cold and temperature abuse conditions. This research would be the first step toward utilizing the survival data of pre-stressed cells when validating food storage conditions. In an effort to mimic the real-world conditions, our work, therefore, provides an assessment of pathogen survival behaviors in refrigeration and temperature abuse models. It was hypothesized that certain preceding hostile conditions may enable *S. enterica* to cope with the environments on baby spinach in cold and temperature abuse scenarios. To the best of our knowledge, this is the first time that the survival of sub-lethally stressed foodborne pathogens on leafy greens during storage has been investigated. We also compared the survival behaviors of pre-stressed *S. enterica* and *E. faecium* NRRL B-2354 on baby spinach under both conditions, which can supplement the dearth of information on the feasibility of using *E. faecium* NRRL B-2354 as a surrogate for *S. enterica* for food storage studies.

## 2. Materials and Methods

### 2.1. Baby Spinach

Conventionally grown baby spinach was purchased from a local grocery store and refrigerated at 4 °C until experiments. To prevent the variability of the raw materials, baby spinach leaves uniform in size and color, without visual defects, were selected. Each sample unit consisted of five baby spinach leaves. Before inoculation, baby spinach was dipped in tap water at 22 °C for 2 min and air-dried at 22 °C for 1 h in a biosafety cabinet.

### 2.2. Strains

*S. enterica* serovars Enteritidis ATCC 13076, Newport ATCC 6962, and Typhimurium ATCC 14028 were used for inoculating baby spinach. *S.* Typhimurium IB43 (*rpoS*::Tn*10d*Cm), a Δ*rpoS* mutant of *S.* Typhimurium ATCC 14028, kindly provided by Ferric C. Fang (University of Washington, Seattle, WA, USA) [20] was used individually to investigate the role of the *rpoS* gene in the persistence of pre-stressed *S. enterica* under cold and temperature abuse conditions. *E. faecium* NRRL B-2354 was also used to determine if it could serve as a non-pathogenic surrogate of *S. enterica*. Spontaneous mutants resistant to 100 µg/mL rifampin were isolated using the gradient plate method [21]. Prior to using rifampin-resistant mutants for subsequent experiments, the survival patterns of the original strains and the mutants on baby spinach under cold and temperature abuse conditions were compared. No significant differences (*p* > 0.05) in the survival behaviors were found between the original strains and the mutants (data not shown). All strains were maintained at −80 °C in tryptic soy broth (TSB) (Fisher Scientific Inc., Hampton, NH, USA) containing 25% (*v*/*v*) glycerol.

### 2.3. Preparation of Inocula

To prepare the inocula, *S. enterica* and *E. faecium* NRRL B-2354 were streaked out from the stock cultures and grown overnight at 37 °C on tryptic soy agar (TSA) (Fisher Scientific Inc.). The cultures were then transferred to TSB supplemented with 100 µg/mL rifampin (TSB-R) and grown overnight at 3 °C. The overnight cultures were washed twice with sterile 0.85% saline. The final pelleted cells were resuspended in 0.85% saline to 9.0 log CFU/mL by measuring the optical density at 600 nm (optical density: 0.7). Equal volumes of *S.* Enteritidis ATCC 13076, Newport ATCC 6962, and Typhimurium ATCC 14028 were then mixed to prepare a cocktail.

### 2.4. Preparation of Pre-Stressed Cells

Both *S. enterica* and *E. faecium* NRRL B-2354 were exposed to sub-lethal desiccation, oxidation, heat shock, or acid stress as follows: (1) Desiccation stress: One milliliter of 1 M NaCl (a_w_ 0.96) containing 9.0 log CFU/mL of bacterial cells was incubated at 22 °C for 2 h [22], (2) Oxidation stress: A mixture of 1 mL of TSB containing 9.0 log CFU/mL of bacterial cells and 1 mL of 300 mg/L sodium hypochlorite (final concentration of sodium hypochlorite: 150 mg/L), as measured by free chlorine test strips (Hach Company, Loveland, CO, USA), was incubated at 22 °C for 2 h [23], (3) Heat shock: One milliliter of TSB containing 9.0 log CFU/mL of bacterial cells was incubated at 48 °C for 1 h [24], and (4) Acid stress: One milliliter of TSB of pH 5.0 by the addition of 1 M hydrochloric acid containing 9.0 log CFU/mL of bacterial cells was incubated at 30 °C for 1.5 h [25]. Fresh cells in sterile 0.85% saline prepared using overnight cultures served as the unstressed control.

After exposure to each sub-lethal stress, cell pellets were washed twice with sterile 0.85% saline and resuspended in 1 mL of sterile 0.85% saline. The population changes in viable cells after each sub-lethal stress were measured by serially diluting in sterile 0.85% saline and spread-plating onto TSA supplemented with 100 µg/mL rifampin (TSA-R), followed by incubation at 37 °C for 24 h.

### 2.5. Inoculation of Baby Spinach

The surface of each baby spinach leaf was spot-inoculated with 100 µL of pre-stressed or unstressed *S. enterica* or *E. faecium* NRRL B-2354 (7.0 log CFU/sample unit) in approximately equal volumes at 20–25 locations, followed by air-drying at 22 °C for 1 h to allow bacterial attachment in the biosafety cabinet.

### 2.6. Storage under Cold and Temperature Abuse Conditions

Afterward, baby spinach was transferred to Ziploc bags and stored at a specified temperature for 7 days as follows: (1) cold (4 °C for 7 days; control) or (2) temperature abuse (37 °C for 2 h, followed by 4 °C for the remainder of the 7-day storage) condition [26]. Samples were collected on days 0, 1, 2, 4, and 7.

### 2.7. Microbiological Analysis

Samples were transferred into individual sterile Whirl-Pak filter bags containing 50 mL of sterile 0.85% saline and homogenized at 260 rpm for 2 min using a Seward stomacher 400 circulator (Seward Ltd., London, UK). Homogenized samples were then serially diluted in sterile 0.85% saline and spread-plated onto TSA-R. Where low levels of viable cells were anticipated, samples were homogenized with 25 mL of sterile 0.85% saline, and 1 mL of undiluted homogenate was plated onto five TSA-R plates with 200 µL for each (detection limit: 1.40 log CFU/sample unit). Plates were incubated at 37 °C for 24 h, and thereafter, bacterial colonies were enumerated.

### 2.8. Mathematical Modeling

To further characterize and quantify the survival behaviors of unstressed and pre-stressed cells, non-linear mathematical models, including the modified Gompertz model, the log-logistic model, the Weibull model, the Geeraerd model, and the biphasic model, were applied to simulate the survival data [27,28,29,30,31,32]. The survival curves were modeled with the Regression Wizard Module in SigmaPlot 14.0 (Systat Software Inc., San Jose, CA, USA). The non-linear regression modeling had the following settings to ensure convergence: iterations = 200; step size = 1; tolerance = 1 × 10^−10^. To evaluate the goodness-of-fit of each model, adjusted *R*^2^ and root mean square error (RMSE) were computed. The closer adjusted *R*^2^ is to 1 and the closer RMSE is to 0, the better the model fits the observations.

### 2.9. Statistical Analysis

All results were obtained from three independent trials. Bacterial counts were expressed as log CFU/sample unit. Analysis of variance (ANOVA), followed by the least-significant-difference (LSD) test, was performed using SigmaPlot 14.0 to assess whether there were significant differences (*p* < 0.05) among treatments.

## 3. Results and Discussion

### 3.1. Survival Behaviors of Pre-Stressed S. enterica

**Figure 1:**
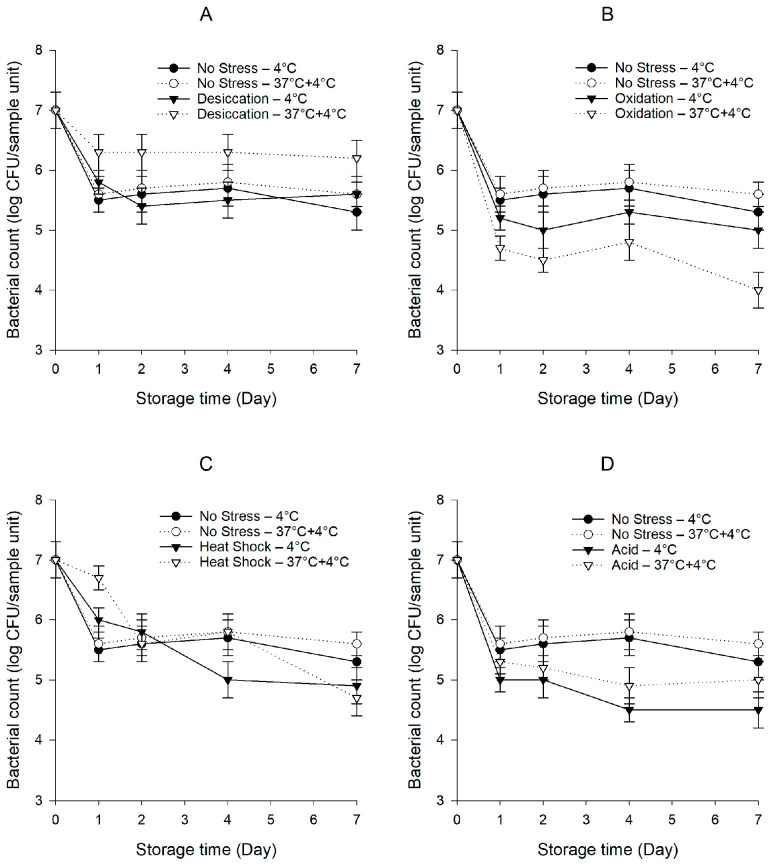
Persistence of the *S. enterica* cocktail on baby spinach under cold and temperature abuse conditions with or without prior exposure to sub-lethal desiccation (**A**), oxidation (**B**), heat shock (**C**), and acid (**D**) stresses. Error bars represent standard deviations (*n* = 3).

After exposure to sub-lethal stresses, the *S. enterica* cocktail declined from 9.0 to 8.4–8.6 log CFU/mL (*p* < 0.05). Figure 1 shows the persistence of the *S. enterica* cocktail on baby spinach with or without previous exposure to sub-lethal stresses under cold and temperature abuse conditions. The bacterial counts of unstressed *S. enterica* were displayed together with those of pre-stressed *S. enterica* for easy comparison. Our study indicates that both unstressed and pre-stressed *S. enterica* involved a pronounced tail in the survival curves. One possible explanation for the tailing is that sensitive cells died at a relatively higher rate at the beginning of the storage, while the cells on the tail with better survival capabilities were left behind. The unstressed *S. enterica* decreased rapidly within the first day and persisted around 5.5 log CFU/sample unit for the remainder of the 7-day storage under both conditions. In the temperature abuse scenario, the unstressed *S. enterica* did not survive better (*p* > 0.05) than under the cold condition.

The desiccation-stressed *S. enterica* did not differ (*p* > 0.05) from unstressed *S. enterica* under the cold condition (Figure 1A). In contrast, our data revealed a clear pattern where the desiccation-stressed *S. enterica* subjected to temperature abuse demonstrated a better persistence capability (*p* < 0.05) compared to under the cold condition. The population of the desiccation-stressed *S. enterica* decreased to 6.3 log CFU/sample unit after the first day and was steadily sustained during the ensuing storage period. The desiccation-stressed *S. enterica* on baby spinach stored under the temperature abuse condition showed the highest bacterial counts (*p* < 0.05) in comparison with the unstressed *S. enterica* and *S. enterica* pre-exposed to other stresses. Interestingly, the unstressed *S. enterica* survived better (*p* < 0.05) than the oxidation- and acid-stressed *S. enterica* under both conditions (Figure 1B,D). Under the temperature abuse condition, the oxidation-stressed *S. enterica* exhibited a steep decline to 4.7 log CFU/sample unit on the first day. This low level was relatively stable in the next 3 days but dropped to 4.0 log CFU/sample unit by the end of the 7-day storage. In contrast, the oxidation-stressed *S. enterica* under the cold condition showed a 0.5–1.0-log higher population (*p* < 0.05) compared to under the temperature abuse condition. During the 7-day storage, the population of the acid-stressed *S. enterica* under the temperature abuse condition was 0.2–0.5 log CFU/sample unit higher (*p* < 0.05) than when the baby spinach was constantly held at 4 °C. Noticeably, there were no significant differences (*p* > 0.05) in the bacterial counts between the unstressed and heat-shocked *S. enterica* (Figure 1C).

The data presented here demonstrate the variation of the responses of *S. enterica*, including cross-protection, sensitization, and no effect, greatly depending on the stress previously applied and the storage condition studied. Our results indicate that desiccation stress led to a pronounced cross-protection of *S. enterica* against circumstances on baby spinach subjected to temperature abuse. It is remarkable that despite the development of enhanced persistence under the temperature abuse condition, desiccation stress did not confer any cross-protection against subsequent survival on baby spinach under the cold condition.

Bacterial cells frequently encounter severe daily or seasonal desiccation in food processing environments, where air and surface dehumidification are performed after daily hygienic operations [33]. Desiccation has been identified as an important factor influencing pathogen survival in the phylloplane as a consequence of the dry condition at the leaf-air boundary, which produces an additional hurdle to bacterial cells on the plant surface [34]. The periodic exposure of bacteria to desiccation stress affects their viability, but consequently, they develop complex strategies to survive and persist. The detailed mechanisms through which prior desiccation stress alters the capacity of bacteria to survive on leaf surfaces are still not well decrypted. The expression of curli, also known as thin aggregative fimbriae (tafi), has been reported to be induced in *S. enterica* when being subjected to desiccation [35]. Several studies have documented the involvement of curli in the persistence and biofilm formation of *S. enterica* on leaf surfaces [36,37]. Our findings demonstrate that *S. enterica*, previously adaptive to desiccation, took greater advantage of the favorable condition at the abusive temperature to resuscitate and persist on baby spinach. One conceivable explanation could be that the biosynthesis of curli in *S. enterica* was inhibited during the cold storage at 4 °C while dramatically increased at the abusive temperature at 37 °C [38,39]. The contamination of leafy greens by desiccation-stressed cells can be a major concern for the food industry due to their extended persistence after being subjected to temperature abuse. It is, thus, critical that processes that involve sub-lethal mild drying be avoided during the production of leafy greens. Additionally, adequate cold storage standards should also be enforced to prevent the occurrence of temperature abuse. These measures will help minimize the incidence of foodborne illnesses caused by sub-lethally stressed cells.

The division ability of *Salmonella* cells can be compromised after exposure to chlorine. In a study by Chaves et al. [40], the first division time of unstressed *S.* Agona was only 1.46 min, whereas it was extended to 19.2 min after being stressed by 150 mg/L sodium hypochlorite for 3 min. The second division time of *S.* Agona stressed by 150 mg/L sodium hypochlorite was still 3.5 times longer than that of untreated cells, indicating that the injury caused by the stress was not fully repaired in the second generation. In the present study, after exposure to 150 mg/L sodium hypochlorite for 2 h, *S. enterica* became impaired in persisting on baby spinach compared to unstressed cells, indicating that the contamination of baby spinach by oxidation-stressed *S. enterica* may not impose an increased risk to the product. These observations could, therefore, be attributed to the longer time required by the cells to repair the sub-lethal injury caused by chlorine. It is worth mentioning here that the oxidation-stressed *S. enterica* subjected to the abusive temperature at 37 °C was more susceptible than when being exposed to constant storage at 4 °C, which could be explained by the inability of oxidation-stressed cells to adjust to frequent temperature fluctuation. Similarly, Norhana et al. [41] have also proved the decreased survival capability of *S*. Senftenberg and Typhimurium exposed to 100 mg/L sodium hypochlorite on shrimp at a higher temperature (25 °C) relative to during cold storage (4 °C). Since chlorine constitutes the most widely used sanitizer for leafy greens in the food industry [42], the quantitative data provided by our research may lead to a better understanding of bacterial responses to chlorine and improved sanitation procedures.

Previous studies have demonstrated the improved viability of heat-shocked foodborne pathogens such as *Cronobacter sakazakii* and *Vibrio parahaemolyticus* at refrigeration temperatures such as 3 and 5 °C [43,44,45]. Conflicting results, however, were obtained in the current study. Irrespective of the storage condition, no enhanced persistence of *S. enterica* on baby spinach was detected following heat shock. Comparing the previously generated data with the ones established in our study will be difficult because of the variations in the microorganisms challenged and the food matrices or models tested. Nevertheless, it can be tentatively speculated that a diverse range of responses to acid stress could be induced in foodborne pathogens following heat shock, depending on the precise experimental conditions involved.

In our research, the *S. enterica* pre-exposed to acid stress was not as persistent as unstressed cells during storage, regardless of the storage condition. The detrimental effect of acidity may have led to the rapid decline in the viable counts of *S. enterica*. Hwang et al. [46] reported that acid treatments at pH 3, 4, and 5 reduced the growth ability of *Listeria monocytogenes* and *Escherichia coli* O157:H7 on cooked ham, which is in general agreement with our findings. Akin to our results that acid-stressed *S. enterica* survived better on baby spinach subjected to temperature abuse than under the cold condition, they also observed that the inhibition effect of acid treatment at pH 3 on the growth of *L. monocytogenes* was reduced at 8 compared to 4 °C. However, the growth rates of acid-stressed *E. coli* O157:H7 at 10 and 12 °C appeared to be similar. Although the exact causes of the differences in the survival behaviors of different foodborne pathogens pre-exposed to acid stress remain to be elucidated, it could be due to the variations in the inherent characteristics of responses that could either enhance, attenuate, or maintain the resistance to the storage scenario.

### 3.2. Survival Behaviors of Pre-Stressed S. Typhimurium ATCC 14028 wild Type and *Δ*rpoS Mutant

**Figure 2:**
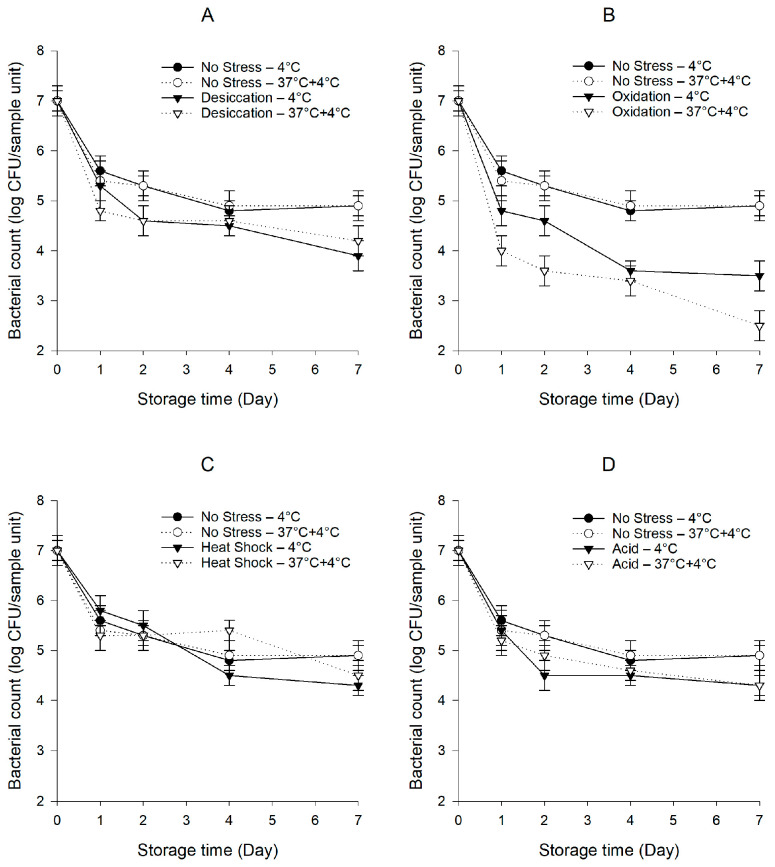
Persistence of *S.* Typhimurium ATCC 14028 wild type and Δ*rpoS* mutant on baby spinach under cold and temperature abuse conditions with or without prior exposure to sub-lethal desiccation (**A**), oxidation (**B**), heat shock (**C**), and acid (**D**) stresses. Error bars represent standard deviations (*n* = 3).

In *Salmonella*, in response to inimical processes used in the food industry, RpoS acts as a master regulator and initiates the transcriptions of a series of genes to protect the cells from being injured or killed [9]. In support of this notion, a previous study by McMeechan et al. [47] showed that using a Δ*rpoS* mutant, RpoS was found to play a dominant role in the long-term survival of *S.* Typhimurium in a saline starvation-survival model at 4.5 °C. It is also generally believed that these response pathways extensively or at least partially overlap, and bacteria exposed to one sub-lethal stress may develop cross-protection against other stresses [4]. To explore the potential involvement of the *rpoS* gene in the persistence of pre-stressed *S. enterica* on baby spinach under cold and temperature abuse conditions, we compared the survival behaviors of pre-stressed *S.* Typhimurium ATCC 14028 wild type with Δ*rpoS* mutant. The populations of Δ*rpoS* mutant decreased from 9.0 to 8.5–8.6 log CFU/mL after being exposed to sub-lethal stresses (*p* < 0.05). *S.* Typhimurium ATCC 14028 wild type and the *S. enterica* cocktail behaved similarly in all cases (*p* > 0.05). Therefore, the data of *S.* Typhimurium ATCC 14028 wild type were not presented in Figure 2. Noticeably, the lack of a fully functional RpoS system resulted in the diminished survival of *S.* Typhimurium ATCC 14028, as the populations of the unstressed and pre-stressed Δ*rpoS* mutant were much lower and decreased more rapidly (*p* < 0.05) compared to those of the unstressed and pre-stressed wild type, respectively. It should be noted that, unlike the wild type, the abusive temperature at 37 °C did not result in the enhanced persistence of the Δ*rpoS* mutant after exposure to desiccation stress (Figure 2A), as the population of the desiccation-stressed Δ*rpoS* mutant was much lower (*p* < 0.05) than that of the unstressed Δ*rpoS* mutant under the temperature abuse condition, suggesting a critical role of the *rpoS* gene in the persistence of the desiccation-stressed *S. enterica* subjected to temperature abuse. Under the temperature abuse condition, the population of the oxidation-stressed Δ*rpoS* mutant was significantly lower (*p* < 0.05) than under the cold condition (Figure 2B). However, compared to the oxidation-stressed wild type, the oxidation-stressed Δ*rpoS* mutant exhibited a much steeper decline (*p* < 0.05) after exposure to the abusive temperature, dropping continuously to 2.5 log CFU/sample unit after 7 days of storage. Similar to the wild type, no significant differences (*p* > 0.05) in bacterial counts were observed between the unstressed and heat-shocked Δ*rpoS* mutant (Figure 2C). The population of the acid-stressed Δ*rpoS* mutant was significantly lower (*p* < 0.05) than that of the unstressed Δ*rpoS* mutant (Figure 2D), while in contrast to the wild type, the acid-stressed Δ*rpoS* mutant survived equally under both conditions (*p* > 0.05).

Abdelhamidab and Yousefac [48] reported the significant expression (>50 fold) of the *rpoS* gene in desiccation-stressed *S.* Tennessee and Eimsbuettel at 4 °C for 2 days. When the storage at 4 °C was extended to 14 days, the up-regulation level of the *rpoS* gene was increased to >300 fold. Ritter et al. [49] observed the active induction of RpoS in *S.* Enteritidis exposed to 200 mg/L sodium hypochlorite, which probably coordinated the expression of other genes involved in stress responses. When Sirsat et al. [50] investigated the transcriptional profile of *S.* Typhimurium subjected to heat shock at 48 °C for 10 min, they observed a 2.75-fold change in the expression of the *rpoS* gene. An early work by Lee et al. [51] reported the involvement of the *rpoS* gene in *S.* Typhimurium for a sustained acid (pH < 4.5) tolerance, as the syntheses of seven acid shock proteins were largely dependent upon RpoS. In the present study, in agreement with the known role of the *rpoS* gene in a general stress response, the deletion mutation of the *rpoS* gene caused a substantially diminished persistence on baby spinach in all cases. Most importantly, desiccation stress could not confer a survival advantage for this mutant subjected to temperature abuse, which further supports the concept that the *rpoS* gene is specifically required for the development of cross-protection by desiccation stress. Altogether, these findings are concurrent with our hypothesis that bacteria can evoke a general stress response through RpoS upon exposure to sub-lethal stresses. Our data, thus, corroborate previous studies investigating the influence of the *rpoS* gene on the prolonged survival of foodborne pathogens. This work helps to advance the mechanistic understanding of bacterial persistence on leafy greens under hostile conditions, where the RpoS system is a key influencing factor. A global transcriptomic analysis using RNA sequencing is needed to deepen the research to dissect the cross-protective responses associated with desiccation stress and the features and/or benefits conferred to bacterial cells.

### 3.3. Survival Behaviors of Pre-Stressed S. enterica and E. faecium NRRL B-2354

**Figure 3:**
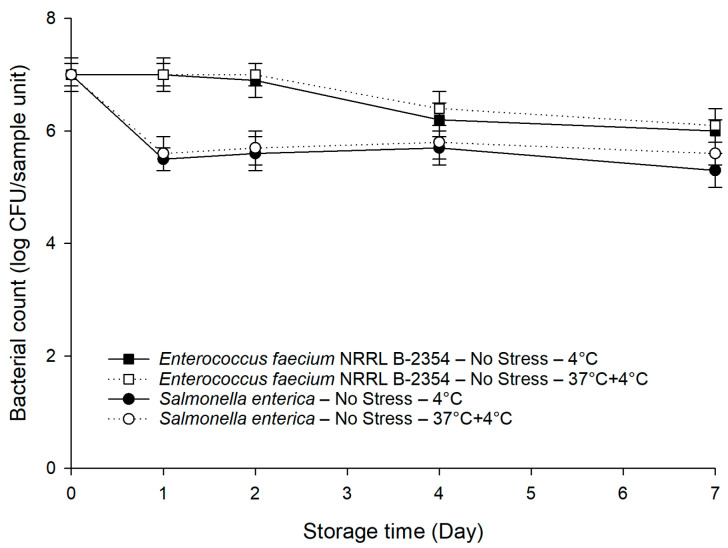
Persistence of *E. faecium* NRRL B-2354 on baby spinach under cold and temperature abuse conditions without prior exposure to sub-lethal stresses compared to a mixture of three *S. enterica* serovars. Error bars represent standard deviations (*n* = 3).

The populations of *E. faecium* NRRL B-2354 subjected to sub-lethal stresses dropped from 9.0 to 8.6–8.7 log CFU/mL (*p* < 0.05). No significant differences (*p* > 0.05) in survival behaviors were observed between the unstressed and pre-stressed *E. faecium* NRRL B-2354 (data not shown). Only the data of the unstressed *E. faecium* NRRL B-2354, as well as the unstressed *S. enterica* cocktail, were shown in Figure 3. As anticipated, the unstressed *E. faecium* NRRL B-2354 had minimal changes within the first 2 days, which was 1.3–1.5 log CFU/sample unit higher (*p* < 0.05) than that of the unstressed *S. enterica* cocktail. After 4 days, there was only a slight decrease in the population of the unstressed *E. faecium* NRRL B-2354 to around 6.0 log CFU/sample unit. The population of the unstressed *E. faecium* NRRL B-2354 was 0.5–0.7 log CFU/sample unit higher (*p* < 0.05) than that of the unstressed *S. enterica* cocktail in the last 3 days of the 7-day storage. We also observed a better persistence capability (*p* < 0.05) of *E. faecium* NRRL B-2354 on baby spinach compared to the pre-stressed *S. enterica* cocktail under cold and temperature abuse conditions, which makes it a logical and conservative surrogate by providing a sufficient safety margin.

*E. faecium* NRRL B-2354 has recently been used as an effective surrogate when investigating the survival of *S. enterica* in low-moisture foods [52,53]. As far as we are aware of, there is still a paucity of evidence regarding the use of *E. faecium* NRRL B-2354 as a surrogate for *S. enterica* on leafy greens during storage. In our study, *E. faecium* NRRL B-2354 was identified as a suitable surrogate for pre-stressed *S. enterica* due to its stable and consistent persistence on baby spinach under cold and temperature abuse conditions. The outputs of this study provide technical information to the food industry for validating the storage conditions for leafy greens in pilot or plant settings.

### 3.4. Mathematical Modeling of the Survival Curves of Pre-Stressed S. enterica and E. faecium NRRL B-2354

The parameter estimates obtained from fitting the experimental observations into five non-linear models, as well as the goodness-of-fit of these models, are presented in Table 1, Table 2, Table 3, Table 4 and Table 5. Overall, the models used in this study were adequate in modeling the survival curves and allowed the fitting of an extended tail for both the unstressed and pre-stressed cells. The good performance of each model was supported by a high average adjusted *R*^2^ (>0.90) and a low average RMSE (<0.50), with the exceptions of only the log-logistic model and the biphasic model that had an average adjusted *R*^2^ of 0.89. Among the five models compared, the modified Gompertz model produced the best overall description to all the survival curves (average adjusted *R*^2^: 0.94; average RMSE: 0.43), closely followed by the Weibull model (average adjusted *R*^2^: 0.92; average RMSE: 0.45) and the Geeraerd model (average adjusted *R*^2^: 0.92; average RMSE: 0.47). The performance of the log-logistic model and the biphasic model was comparable, both yielding an average adjusted *R*^2^ of 0.89 and an average RMSE of 0.46.

The modified Gompertz model was developed primarily to model the asymmetrical sigmoidal shape of microbial growth curves [31]. As characterized by the greatest *M* and the lowest *C* in the modified Gompertz model, the more robust persistence of *E. faecium* NRRL B-2354 turned out in survival curves with the longest shoulders and the smallest differences in the upper and lower asymptotes under cold and temperature abuse conditions. The survival curve of *E. faecium* NRRL B-2354 also had the lowest relative death rate at *M* under the cold condition, as indicated by *B*. The survival curves of the desiccation-stressed *S. enterica* cocktail also had a lower *C* than those of the unstressed, oxidation-stressed, heat-shocked, and acid-stressed *S. enterica* under both conditions. Compared to the wild type, the desiccation- and oxidation-stressed Δ*rpoS* mutant required shorter times to reach the maximum absolute death rate and their survival curves had greater differences in the upper and lower asymptotes.

The log-logistic model was proposed by Cole et al. [29] to describe the non-linear thermal inactivation of microbes and was simplified by Chen and Hoover [28]. The highest *A* in the log-logistic model was obtained from simulating the survival curves of *E. faecium* NRRL B-2354 under cold and temperature abuse conditions, demonstrating the smallest differences in the upper and lower asymptotes, which is in agreement with the curve fitting using the modified Gompertz model. The desiccation-stressed *S. enterica* cocktail needed longer times to reach the maximum death rate (greater *τ*) and had smaller differences in the upper and lower asymptotes under the temperature abuse condition than under the cold condition. In contrast, a lower *τ* and a lower *A* were found in the oxidation-stressed *S. enterica* cocktail under the temperature abuse condition than under the cold condition. A lower *τ* for the Δ*rpoS* mutant suggested that shorter times were required to reach the maximum death rate compared to the wild type in most cases. The desiccation- and oxidation-stressed Δ*rpoS* mutants had greater differences in the upper and lower asymptotes compared to the wild type.

The Weibull model assumes that cells in a population have different resistance levels and considers survival curves as a cumulative form for the distribution of lethal events [32]. The most obvious deficiency of the Weibull model is the lack of well-defined biological meanings of the parameters. Nonetheless, a link can still be established between the parameters of the Weibull model and the shape of the survival curve. Downward concavity (*τ* > 1) indicates that cells are more resistant at the beginning (a long shoulder), whereas upward concavity (*τ* < 1) indicates that cells can gradually adapt to the environment over time (a long tail) [32]. In this study, *τ* was <1 in most cases, suggesting survival curves with an upward concavity, except for the survival curves of *E. faecium* NRRL B-2354 with a better persistence capability that showed longer shoulders. However, it should be remarked that *τ* exhibited no dependencies on either microorganism, storage condition, or stress type, and it is, therefore, difficult to draw a definite conclusion from this parameter.

The Geeraerd model can exhibit a log-linear behavior with and without shoulder and/or tailing revealing a smooth transition between each phase [30], in which the tailing phase is considered for a population remaining constant in time or not suffering any significant succeeding reduction. Our curve fitting using the Geeraerd model showed that the unstressed *S. enterica* cocktail under cold and temperature abuse conditions had the highest maximum specific death rates (highest *k*_max_), while the highest residual population densities (highest *N*_res_) were obtained from the desiccation-stressed *S. enterica* cocktail under the temperature abuse condition. The pre-stressed wild type had a higher *N*_res_ than the pre-stressed Δ*rpoS* mutant in nearly all cases, which reflected a higher number of viable cells remaining at the end of storage. It is interesting to note that a higher *k*_max_ was also observed in the pre-stressed wild type compared to the pre-stressed Δ*rpoS* mutant. Only one exception existed; the heat-shocked wild type had a lower residual population density (lower *N*_res_) and maximum specific death rate (lower *k*_max_) than the pre-stressed Δ*rpoS* mutant under the temperature abuse condition. Although with the lowest *k*_max_, *E. faecium* NRRL B-2354 had an erratically lower *N*_res_, which could be attributed to the inadequate curve fitting using the Geeraerd model due to their longer shoulders, as indicated by the relatively lower average adjusted *R*^2^.

Regarding the biphasic model, the biphasic feature of a survival curve indicates the presence of heterogeneous bacterial populations concerning their sensitivity levels [27]. The curve can be interpreted as containing two sub-populations that decay independently according to the log-linear kinetics. The tailing of the curve occurs due to the different susceptibilities of the cells or the presence of protective features within the ecosystem. The first portion of the curve describes the death of the least resistant (sensitive) cells and the second portion (tail), the death of the most resistant cells. A pronounced tailing effect was further confirmed by our curve fitting using the biphasic model, as suggested by the lower *k*_max2_ (death rate of the resistant population) compared to the *k*_max1_ (death rate of the sensitive population). Tailing is also defined in the biphasic model through the parameter *f*, whose value corresponds to the proportion of the sensitive population, and where (1-*f*) represents the proportion of the resistant population. The parameter estimates for *E. faecium* NRRL B-2354 with a lower adjusted *R*^2^ and higher RMSE, possibly owing to the lack of a sensitive sub-group in its population, were not considered. The curve fitting using the biphasic model yielded an *f* that ranged from 0.78 to 1.00. Our findings implicate that cells from the tail should be sufficiently considered, as inadequate postharvest sanitation or improper storage would lead to the survival of resistant *S. enterica* cells on baby spinach. The unstressed *S. enterica* cocktail had a much higher death rate in the sensitive population than the pre-stressed *S. enterica* cocktail. Noticeably, a higher *k*_max1_ was observed in the pre-stressed *S. enterica* cocktail and Δ*rpoS* mutant under the temperature abuse condition compared to under the cold condition, with acid-stressed cells as the only exception, indicating that a population of pre-stressed cells was less tolerant to temperature abuse. However, the parameters of the biphasic model did not exhibit any dependencies on stress type.

Our study lays the groundwork for taking pre-stressed cells into consideration to improve the challenge studies that can be developed for other pathogen-food combinations, which is critical for making risk assessment decisions and protecting public health. From a practical point of view, since dehumidification is used on a daily basis after cleaning–disinfection procedures in the food industry, desiccation-stressed *S. enterica* could constitute a risk to the microbial safety of leafy greens. The data generated from our challenge studies with pre-stressed cells can also be used to increase the accuracy of predictive models and lead to the future development of reliable quantitative risk assessment schemes, which can facilitate the establishment of food safety objectives and be implemented in hazard analysis critical control point (HACCP) systems to enhance microbial food safety. Nevertheless, cautions should be exercised in interpreting these results, as it is still unknown if the data reported here can be generalized to other types of leafy greens and more complicated temperature fluctuations.

## 4. Conclusions

The results established in the current study contribute to the knowledge on the effects of stresses encountered by *S. enterica* in the food production systems on their persistence during subsequent storage. Our observations strongly support that the influence of prior stress on the survival characteristics of *S. enterica* on leafy greens during storage may vary, which is largely dependent upon the stress type and the storage condition. This fact, together with the survival kinetics described using various non-linear models, indicates that there should be great diversity within the mechanisms of these stresses.

Future research is needed to develop more complex stressful circumstances encountered in the food production systems to elucidate the impact of stress-hardening on foodborne pathogens with a nexus to food safety and risk assessment. Studies on the possible difference in sensitivity between planktonic cells and more recalcitrant biofilms are also merited. Additionally, understanding the molecular events jointly involved in the cross-protective responses of pre-stressed foodborne pathogens to cold and temperature abuse conditions will provide valuable information.

## Figures and Tables

**Table 1:** Parameter estimates and goodness-of-fit of the modified Gompertz model for the persistence of *S. enterica* and *E. faecium* NRRL B-2354 on baby spinach under cold and temperature abuse conditions with or without prior exposure to sub-lethal stresses.

Sub-Lethal Stress	Microorganism	Storage Condition	Parameter Estimate ^a^			Goodness-of-Fit	
			*M*	*B*	*C*	Adjusted *R*^2^	RMSE ^b^
No stress	*S. enterica* cocktail	Cold	−0.58	12.43	1927.91	0.90	0.46
		Temperature abuse	−0.38	14.57	320.15	0.96	0.35
	*S.* Typhimurium IB43 ^c^	Cold	−0.18	1.26	3.75	0.99	0.24
		Temperature abuse	−3.70	1.59	732.10	0.96	0.41
	*E. faecium* NRRL B-2354	Cold	3.65	−1.37	−1.01	0.99	0.10
		Temperature abuse	3.56	2.05	0.90	1.00	0.00
Desiccation	*S. enterica* cocktail	Cold	0.93	20.00	1.50	0.98	0.32
		Temperature abuse	−1.38	2.96	43.61	0.97	0.24
	*S.* Typhimurium IB43	Cold	−6.11	0.90	697.56	0.90	0.61
		Temperature abuse	−1.92	2.93	648.54	0.96	0.46
Oxidation	*S. enterica* cocktail	Cold	−1.99	3.07	862.80	0.96	0.41
		Temperature abuse	−2.48	2.17	556.95	0.88	0.63
	*S.* Typhimurium IB43	Cold	−7.07	0.80	997.60	0.94	0.58
		Temperature abuse	−4.60	1.20	997.70	0.92	0.70
Heat shock	*S. enterica* cocktail	Cold	−9.22	0.50	218.16	0.96	0.42
		Temperature abuse	−30.15	0.18	628.21	0.75	0.68
	*S.* Typhimurium IB43	Cold	−9.64	0.46	236.44	0.97	0.45
		Temperature abuse	−3.71	1.66	943.12	0.72	0.69
Acid	*S. enterica* cocktail	Cold	−4.00	1.50	966.62	0.95	0.49
		Temperature abuse	−3.31	1.75	657.42	0.98	0.36
	*S.* Typhimurium IB43	Cold	0.71	2.48	2.61	0.99	0.32
		Temperature abuse	−5.25	1.09	777.00	0.97	0.44
Average						0.94	0.43

^a^*M*, time to reach the maximum absolute death rate (day); *B*, relative death rate at *M* (log CFU/sample unit); *C*, difference in the values of the upper and lower asymptotes (log CFU/sample unit). ^b^ RMSE, root mean square error. ^c^ *S.* Typhimurium IB43 is the Δ*rpoS* mutant of *S.* Typhimurium ATCC 14028.

**Table 2:** Parameter estimates and goodness-of-fit of the log-logistic model for the persistence of *S. enterica* and *E. faecium* NRRL B-2354 on baby spinach under cold and temperature abuse conditions with or without prior exposure to sub-lethal stresses.

Sub-Lethal Stress	Microorganism	Storage Condition	Parameter Estimate ^a^			Goodness-of-Fit	
			A	σ	τ	Adjusted R^2^	RMSE ^b^
No stress	*S. enterica* cocktail	Cold	−3.33	−0.23	1.14	0.82	0.53
		Temperature abuse	−3.10	−0.18	1.38	0.86	0.47
	*S.* Typhimurium IB43 ^c^	Cold	−2.08	−2.60	−0.10	0.98	0.33
		Temperature abuse	−3.91	−0.60	0.49	0.99	0.26
	*E. faecium* NRRL B-2354	Cold	−1.02	−2.97	0.49	1.00	0.00
		Temperature abuse	−0.90	−17.12	0.59	1.00	0.00
Desiccation	*S. enterica* cocktail	Cold	−3.25	−0.24	1.18	0.83	0.52
		Temperature abuse	−2.34	−0.23	2.43	0.93	0.28
	*S.* Typhimurium IB43	Cold	−4.90	−1.17	0.56	0.84	0.69
		Temperature abuse	−4.79	−0.34	0.59	0.91	0.56
Oxidation	*S. enterica* cocktail	Cold	−3.88	−0.27	0.54	0.89	0.53
		Temperature abuse	−4.59	−0.59	0.09	0.82	0.70
	*S.* Typhimurium IB43	Cold	−5.15	−1.76	0.26	0.94	0.59
		Temperature abuse	−5.67	−1.40	−0.02	0.90	0.74
Heat shock	*S. enterica* cocktail	Cold	−3.54	−1.54	0.57	0.91	0.51
		Temperature abuse	−345.17	−137.84	3.99	0.53	0.79
	*S.* Typhimurium IB43	Cold	−4.69	−2.11	0.63	0.93	0.54
		Temperature abuse	−4.69	−0.74	1.09	0.66	0.73
Acid	*S. enterica* cocktail	Cold	−4.28	−0.72	0.28	0.96	0.46
		Temperature abuse	−3.90	−0.44	0.55	0.97	0.40
	*S.* Typhimurium IB43	Cold	−2.61	−5.43	−0.06	0.99	0.37
		Temperature abuse	−3.75	−1.10	0.08	1.00	0.22
Average						0.89	0.46

^a^*A*, difference in the values of the upper and lower asymptotes (log CFU/sample unit); *τ*, log time to reach the maximum death rate (log day); *σ*, maximum death rate at *τ* [log (CFU/sample unit)/log day]. A small *t_0_* (10^−6^) was used instead of 0 since log*t_0_* at *t_0_* = 0 was not defined. ^b^ RMSE, root mean square error. ^c^ *S.* Typhimurium IB43 is the Δ*rpoS* mutant of *S.* Typhimurium ATCC 14028.

**Table 3:** Parameter estimates and goodness-of-fit of the Weibull model for the persistence of *S. enterica* and *E. faecium* NRRL B-2354 on baby spinach under cold and temperature abuse conditions with or without prior exposure to sub-lethal stresses.

Sub-Lethal Stress	Microorganism	Storage Condition	Parameter Estimate ^a^		Goodness-of-Fit	
			*σ*	*τ*	Adjusted *R*^2^	RMSE ^b^
No stress	*S. enterica* cocktail	Cold	52,015,161.74	0.14	0.89	0.47
		Temperature abuse	63,185,071.76	0.14	0.86	0.47
	*S.* Typhimurium IB43 ^c^	Cold	18,160.77	0.23	0.94	0.46
		Temperature abuse	578,627.64	0.16	1.00	0.20
	*E. faecium* NRRL B-2354	Cold	74.27	1.08	0.81	0.46
		Temperature abuse	56.14	1.30	0.85	0.40
Desiccation	*S. enterica* cocktail	Cold	52,578,779.99	0.14	0.89	0.47
		Temperature abuse	37,964,061.33	0.19	0.92	0.30
	*S.* Typhimurium IB43	Cold	524.37	0.33	0.94	0.44
		Temperature abuse	55,720,998.56	0.10	0.95	0.48
Oxidation	*S. enterica* cocktail	Cold	60,438,737.10	0.12	0.92	0.48
		Temperature abuse	528,654.13	0.14	0.91	0.58
	*S.* Typhimurium IB43	Cold	185.32	0.35	0.97	0.50
		Temperature abuse	140.04	0.29	0.98	0.50
Heat shock	*S. enterica* cocktail	Cold	346.91	0.46	0.94	0.45
		Temperature abuse	72.05	0.77	0.76	0.67
	*S.* Typhimurium IB43	Cold	125.13	0.52	0.96	0.48
		Temperature abuse	17,915.52	0.23	0.84	0.61
Acid	*S. enterica* cocktail	Cold	104,874.62	0.17	0.98	0.39
		Temperature abuse	31,215,549.32	0.12	0.98	0.33
	*S.* Typhimurium IB43	Cold	2179.80	0.26	0.92	0.56
		Temperature abuse	3360.39	0.25	1.00	0.10
Average					0.92	0.45

^a^*σ*, scale factor; *τ*, shape factor. ^b^ RMSE, root mean square error. ^c^ *S.* Typhimurium IB43 is the Δ*rpoS* mutant of *S.* Typhimurium ATCC 14028.

**Table 4:** Parameter estimates and goodness-of-fit of the Geeraerd model for the persistence of *S. enterica* and *E. faecium* NRRL B-2354 on baby spinach under cold and temperature abuse conditions with or without prior exposure to sub-lethal stresses.

Sub-Lethal Stress	Microorganism	Storage Condition	Parameter Estimate ^a^		Goodness-of-Fit	
			*N*_res_	*k*_max_	Adjusted *R*^2^	RMSE ^b^
No stress	*S. enterica* cocktail	Cold	5.53	35.33	0.90	0.46
		Temperature abuse	5.68	39.52	0.96	0.35
	*S.* Typhimurium IB43 ^c^	Cold	4.93	1.02	0.99	0.26
		Temperature abuse	4.95	1.58	0.96	0.41
	*E. faecium* NRRL B-2354	Cold	3.22	0.05	0.81	0.46
		Temperature abuse	−305.01	0.00	0.83	0.42
Desiccation	*S. enterica* cocktail	Cold	5.50	1.79	0.96	0.36
		Temperature abuse	6.26	2.95	0.97	0.24
	*S.* Typhimurium IB43	Cold	4.21	0.89	0.90	0.61
		Temperature abuse	4.67	2.92	0.96	0.46
Oxidation	*S. enterica* cocktail	Cold	5.10	3.07	0.96	0.41
		Temperature abuse	4.42	2.17	0.88	0.63
	*S.* Typhimurium IB43	Cold	3.53	0.78	0.94	0.57
		Temperature abuse	2.98	1.19	0.92	0.70
Heat shock	*S. enterica* cocktail	Cold	4.79	0.49	0.96	0.42
		Temperature abuse	3.82	0.17	0.75	0.68
	*S.* Typhimurium IB43	Cold	4.14	0.45	0.97	0.45
		Temperature abuse	5.01	1.65	0.72	0.69
Acid	*S. enterica* cocktail	Cold	4.59	1.49	0.95	0.49
		Temperature abuse	5.00	1.74	0.98	0.36
	*S.* Typhimurium IB43	Cold	4.33	1.03	0.98	0.41
		Temperature abuse	4.46	1.08	0.97	0.44
Average					0.92	0.47

^a^*k*_max_, maximum specific death rate (1/day); *N*_res_, residual population density (log CFU/sample unit). ^b^ RMSE, root mean square error. ^c^ *S.* Typhimurium IB43 is the Δ*rpoS* mutant of *S.* Typhimurium ATCC 14028.

**Table 5:** Parameter estimates and goodness-of-fit of the biphasic model for the persistence of *S. enterica* and *E. faecium* NRRL B-2354 on baby spinach under cold and temperature abuse conditions with or without prior exposure to sub-lethal stresses.

Sub-Lethal Stress	Microorganism	Storage Condition	Parameter Estimate ^a^			Goodness-of-Fit	
			f	K_max1_	K_max2_	Adjusted R^2^	RMSE ^b^
No stress	*S. enterica* cocktail	Cold	0.96	3,836,776.52	0.08	0.87	0.50
		Temperature abuse	0.95	432,929.63	0.01	0.92	0.41
	*S.* Typhimurium IB43 ^c^	Cold	0.99	3.12	0.04	0.97	0.39
		Temperature abuse	0.98	86.24	0.19	1.00	0.20
	*E. faecium* NRRL B-2354	Cold	1.00	0.48	−2.06	0.71	0.51
		Temperature abuse	−19.12	0.24	0.24	0.66	0.50
Desiccation	*S. enterica* cocktail	Cold	0.98	3.19	−0.10	1.00	0.17
		Temperature abuse	0.78	15,146.79	0.04	0.98	0.20
	*S.* Typhimurium IB43	Cold	0.99	4.47	0.35	0.93	0.56
		Temperature abuse	0.99	3383.31	0.08	0.93	0.53
Oxidation	*S. enterica* cocktail	Cold	0.98	2291.49	0.04	0.91	0.50
		Temperature abuse	0.99	6949.39	0.23	0.87	0.64
	*S.* Typhimurium IB43	Cold	0.99	5.96	0.47	0.90	0.67
		Temperature abuse	1.00	29.79	0.54	0.99	0.45
Heat shock	*S. enterica* cocktail	Cold	0.98	1.59	0.08	0.87	0.55
		Temperature abuse	0.80	1.99	0.51	0.58	0.77
	*S.* Typhimurium IB43	Cold	0.99	1.89	0.16	0.90	0.59
		Temperature abuse	0.96	1367.09	0.30	0.80	0.64
Acid	*S. enterica* cocktail	Cold	0.99	68.38	0.22	0.94	0.49
		Temperature abuse	0.98	5.09	0.08	0.96	0.42
	*S.* Typhimurium IB43	Cold	1.00	3.88	0.08	0.99	0.30
		Temperature abuse	0.99	5.09	0.27	1.00	0.22
Average						0.89	0.46

^a^*f*, fraction of the sensitive population; *K*_max1_, death rate of the sensitive population (1/day); *K*_max2_, death rate of the resistant population (1/day). ^b^ RMSE, root mean square error. ^c^ *S.* Typhimurium IB43 is the Δ*rpoS* mutant of *S.* Typhimurium ATCC 14028.

## Data Availability

Authors will make data available upon request.
